# Predictors and prevalence of stress, anxiety, depression, and PTSD among university students during the second wave of the COVID-19 pandemic in Turkey

**DOI:** 10.3389/fpsyg.2022.1087528

**Published:** 2023-01-10

**Authors:** Imran Aslan, Orhan Çınar

**Affiliations:** ^1^Faculty of Health Sciences, Health Management Department, Bingöl University, Bingöl, Türkiye; ^2^Faculty of Economics and Administrative Sciences, Atatürk University, Erzurum, Türkiye

**Keywords:** depression, anxiety, PTSD—posttraumatic stress disorder, stress, COVID-19, students, university

## Abstract

This study aimed to find the prevalence of stress, anxiety, depression, and PTSD; differences according to demographic variables; and predictors of mental health problems during the second wave of the coronavirus disease (COVID-19) pandemic in Turkey. Differences in exposure to COVID-19 during the first and second waves of the pandemic among students were compared. A total of 754 students from seven universities in different parts of Turkey participated in the survey between November and December 2020. Perceived Stress Scale (PSS-10), Generalized Anxiety Disorder (GAD-7), Patient Health Questionnaire (PHQ-9), Checklist-Civilian Version (PCL-C) scale measuring posttraumatic stress disorder (PTSD), and Satisfaction with Life Scale (SWLS) were used to measure the mental well-being of students. Descriptive statistics, one-way ANOVA, correlations, and multinomial logistic regression methods were used to analyze the data. The prevalence of high stress, high generalized anxiety (GAD-7 ≥ 10), high depression symptoms (PHQ-9 ≥ 10), and high PTSD in the total sample were 84.2, 36.2, 55.0, and 61.2%, respectively. High perceived stress, moderate generalized anxiety disorder, mild depression symptoms, high severity PTSD, and moderate satisfaction were found among students in Turkey. Religiosity and spirituality have significant negative correlations with anxiety, depression, and PTSD. Religiosity level, gender, relationship status, year of study, physical activity, symptoms of coronavirus, death of a close relative, job loss, and economic status are significant parameters for predicting psychological problems of students in Turkey.

## 1. Introduction

The coronavirus disease (COVID-19) is a highly infectious disease and has affected millions of people globally (Deng et al., 2021). Quarantine or social isolation, worsening economic situations, business closures, distance education, increasing unemployment, concerns about the present and future, deaths, and increasing number of cases were some major challenges that disrupted people’s lives (Dagnino et al., 2020; Chaturvedi et al., 2021) and increased distress, loneliness, insomnia, anxiety, and depression prevalence, leading to some physical and mental health problems (Ahmed et al., 2020; Lee et al., 2021). The most serious problem was the increasing number of new cases and deaths, resulting in more fear among people as many had lost their family members and close friends, and they had a persistent fear of losing more members or getting infected. Much of the information distribution about the pandemic has occurred on social media platforms (Facebook, Twitter, Instagram, etc.) as essential sources of information during the pandemic due to social distancing rules, lockdowns, and strict quarantine measures. Media coverage and social media discourses, such as misinformation and infodemics, and unverified rumors about uncertainty over the disease’s status have increased people’s fear. However, alerting the public, increasing awareness, spreading knowledge and news, connecting people to get helps, and promoting certain preventive behaviors among the public were positive roles of social media (Chen et al., 2020; Cinelli et al., 2020; Kadam and Atre, 2020; Yassin et al., 2021).

This study was carried out among university students between November and December 2020 in Turkey. At the end of December 2020, there were about 15,000 new cases per day and 20,884 total deaths according to Worldometers (2021) statistics. The first confirmed case of COVID-19 in Turkey was reported on March 10, 2020. Closing schools and universities; physical or social distancing; quarantining; closing non-essential shops, restaurants, gyms, sport facilities, and theaters; banning all social gatherings; covering coughs and sneezes; hand washing and keeping unwashed hands away from the face; use of face masks; practicing good respiratory hygiene; and limiting traveling and socialization were some measures taken to minimize the risk of transmissions by governments during the pandemic in Turkey (Küçükali and Çınar, 2020).

Well-being is explained by less stress, optimism, self-esteem, higher life satisfaction, and the development of positive relationships with a state of physical, mental, spiritual, and social integration (Braun et al., 2020; Kilani et al., 2020). High stress, fear, worries, anxiety, and depression as main threats of well-being have increased to a dangerous level mainly due to social isolation related to restrictions, mandatory curfews, and layoffs due to financial difficulties and economic problems in Turkey (Aslan et al., 2020; Kikuchi et al., 2020; Chaturvedi et al., 2021; Ahmed et al., 2022). Higher education students were vulnerable to developing mental health disorders during this pandemic due to academic pressure, losing track of studies and assignments, financial difficulties, and deviations from their everyday routines (Aslan, 2021a; Deng et al., 2021); high mental health deterioration has been seen among young adults (Elmer et al., 2020; Liang et al., 2020; Fried et al., 2022). Fear of infections, fear of losing relatives, and anxiety were found as the most serious responses during the COVID-19 pandemic among students (Aslan, 2021a). The students were mostly affected by the lockdowns (Matthewman and Huppatz, 2020), leading to depression, substance use, difficulties in sleeping disorders, stress, and mal eating habits (Hidayu and Vasudevan, 2020; Smith et al., 2020). In the study by Aslan et al. (2020), distance learning, increasing unemployment, more challenging career opportunities, obeying lockdown rules, decreased activities, financial situation, and completion of the semester were major negative factors affecting the well-being of students in Turkey. Higher levels of stress, depression, anxiety disorder, posttraumatic stress disorder (PTSD), and lower satisfaction were seen among university students under the conditions of the COVID-19 pandemic (Aslan et al., 2020; Ye et al., 2020; Aslan, 2021a). Students were more vulnerable to depression compared to other population groups as their mental health issues can decrease employment opportunities and result in low academic outcomes and earning opportunities in the future. It is aimed in this quasi-experimental design study to disclose the prevalence and predictors of mental health among university students during the second wave of the COVID-19 pandemic and to check whether there were improvements in their mental well-being compared to the first wave (April–May 2020) period in Turkey. Different from the first wave, the degree of associations between exposure to COVID-19 during the second wave and the risk of coronavirus-related PTSD was to be uncovered, as COVID-19 can turn out to become a significant risk for students in the long run. Moreover, relationships of religiosity and spirituality with mental health problems were determined to measure the resilience of students. Unlike the first study, a question about the students’ intention to die by suicide was added. Students from seven universities in different parts of Turkey were surveyed in this study. This article assumes that the female gender, living in a rural area, higher education level, and lower religiosity and spirituality are risk factors for all measured mental health dimensions.

## 2. Literature review

High perceived stress, generalized anxiety disorder, depression, and PTSD have been seen among students during the pandemic. Perceived stress, defined by the level of symptoms of relaxation difficulty, nervous stimulation, quick worry, pressure, discomfort, overreaction, and intolerance (Doğan and Doğan, 2019), is an imbalance between an individual’s perception and external demands. Generalized anxiety disorder (GAD) is described by sadness, fear (Quek et al., 2019), persistence of excessive worries, distressing emotions, physiological arousal, bodily sensations, thoughts of danger avoidance and other defensive behaviors, and nervousness (Spitzer et al., 2006). Uncertainty, diminished medical access, isolation due to social distancing, and family relations (e.g., family concerns and domestic violence) were the causes of the deterioration of anxiety (Smith et al., 2020). People with high anxiety made hospitals crowded by going to physicians frequently for testing and controls around the world that they may be infected. High anxiety responses among students were seen due to not having a cure and vaccine. Also, the effects of COVID-19 on their studies, such as distance education and lockdowns, led to higher anxiety (Quek et al., 2019). Depression is defined by a feeling of worthlessness, dissatisfaction, despair, loss of interest, and low energy (Doğan and Doğan, 2019). People with a lack of social interactions have more tendency to depression (Shafiq et al., 2021). The depression could be related both directly and positively to the fear of COVID-19 and stress, and indirectly and positively mediated to anxiety during the lockdown among undergraduates (Rodríguez-Hidalgo et al., 2020). PTSD is defined as an uncontrollable thought process about the event, unwanted distressing memories of the traumatic event, sleeping and concentrating problems, flashbacks, nightmares, memory problems, and a lack of interest in activities (Kirkpatrick and Heller, 2014). Factors associated with increased levels of depression, anxiety, stress, and PTSD include an increase in time spent on social media, TV and movies, and sleep duration and a decrease in physical activities (Adewale et al., 2021).

From the studies conducted during the COVID-19 pandemic, it was found that limited resources, losing track of their studies, disruptions of relationships, fear of the COVID-19 (Hidayu and Vasudevan, 2020), presence of someone hospitalized for the COVID-19 in one’s household, reduced learning time, conflicts at home and with neighbors, difficulties of the isolation, noise inside or outside one’s home, the perceived ineffectiveness of the use of media entertainment (Bourion-Bédès et al., 2021), preexisting health conditions (Kim et al., 2020), lack of access to technology (Jawad et al., 2020), need for a quiet place to study, home duties, efforts in taking care of siblings (Hoyt et al., 2020), limited class interaction and inefficient time (Chaturvedi et al., 2021), decrease in family income, lack of media access (Jawad et al., 2020), social difficulties and lack of interpersonal communication (AlAteeq et al., 2020; Chaturvedi et al., 2021), rumors, panic, the unpredictability and uncertainty of the situation (AlAteeq et al., 2020), longer quarantine duration, infection fears, frustration, boredom (Shafiq et al., 2021), rumination focusing on negative emotions (Ye et al., 2020), decreased motor activities, increased alcohol use and tobacco consumption, shifts in the food habits, less exposure to sunlight, and physical distancing (Bourion-Bédès et al., 2021; Ahmed et al., 2022) were some sources of stress affecting students well-being. Moreover, current studies and future career worries, non-opening of educational institutions, difficulties with the payment of tuition fees in India (Chhetri et al., 2021), academic difficulties (AlAteeq et al., 2020), worries about semester and graduation completion, being afraid of not finding a job after graduation due to a profession’s lack of knowledge and professional skills (Valero-Chillerón et al., 2019), and worsening relationships (Aslan, 2021a) are some other sources of excessive stress among students. Also, in another study (Jawad et al., 2020), it was stated that many students have got graduated even without proper training and exams and that many students think that they may not be successful after graduation exams and their future will be affected due to detrimental effects on their performance. However, COVID-19 disruptions have caused some positive results besides challenges and drawbacks. Implementation of online learning, socialization opportunities by enhancing social interaction during virtual learning, being able to get online emotional and psychological support (Kee, 2021), improving online learning skills (Kumpikaitė-Valiūnienė et al., 2021), connecting strongly with family members, relating more to spirituality and religion as the appreciation of life, caring better for the environmental and personal hygiene, social unity and strengthening the connectedness of communities (Alghamdi, 2021), improving resilience against crises (Aslan, 2021a), raising compassion, and take more time to yourself are some perceived positive outcomes. Being continuously exposed to stressors can cause more serious mental problems such as depression, PTSD, and even suicide intentions in the long-term that people cannot face this stress anymore, leading to exhaustion, low energy, and mental fatigue (Membrive-Jiménez et al., 2020; Supervía and Bordás, 2020). Traumatic cases like the death of a relative, sexual assault, warfare, traffic collision, and threats to a person’s life can cause PTSD (Bridgland et al., 2021; Menon et al., 2021). The students’ PTSD symptoms were significantly predicted by family members suspected of COVID-19 or who died from COVID-19 (Li et al., 2021). This unconventional grieving process could deeply traumatize people and leave an unhealed psychological trauma for a long time. The lack of social support and the breakdown of social support structures due to the loss of loved ones were strong predictors of PTSD. Decreased life quality, loss of life satisfaction as low happiness and sense of worthwhile increasing the risk of transmitting the disease, more worries as “nothing will be the same” due to changes in lifestyles (sleep disruption, altered eating habits, and reduced physical activity) during the pandemic as stated in Caroppo et al.’s (2021) study, increased concerns about the future, and questioning the meaning of life as signs of posttraumatic growth levels are some consequences of COVID-19 (Fujiwara et al., 2020; Wright et al., 2020). Drug abuse, depression, and sleep problems can be developed if PTSD symptoms are not treated properly (Kirkpatrick and Heller, 2014; Alshehri et al., 2020). Psychological distress can decrease self-esteem or self-efficacy and increase mental illnesses and suicidal ideations (Deng et al., 2021); globally, 90% of the suicide occurrences take place in extreme cases (Song et al., 2020). For example, economic loss, a significant risk for PTSD, is estimated to be a reason for increased suicides in Japan (Fujiwara et al., 2020; Kikuchi et al., 2020).

An increase in stress and anxiety and a slight decrease in depression were measured among Bingöl University students at the end of 2020 in Turkey. The slight decrease in depression could be explained by decreased panic and getting used to the distance education and situation (Aslan, 2021b). The experience of early life adversity (being neglected and abused) and exposure to traumas increasing psychological distress from a study applied to university students in China are risk factors for mental problems during the COVID-19 pandemic (Li et al., 2021). Moderate levels of perceived stress and anxiety during the pandemic were found, and 35.6% of students stated that they have emotional distress due to the COVID-19 pandemic (Hoyt et al., 2020). High stress, anxiety, and depression with lower well-being were found among students during the COVID-19 in Bangladesh, French, Pakistan, the United States of America (USA), and China (Jawad et al., 2020; Ahmed et al., 2022); 22% of French students had a prevalence of severe perceived stress (Bourion-Bédès et al., 2021), and 15% of the students had moderately severe depression during the pandemic in Bangladesh (Islam et al., 2020). A pooled depressive symptoms prevalence of 36% and anxiety symptoms prevalence of 32% were found among higher education students (Deng et al., 2021). The prevalence of PTSD among infected students was 27.1% (Li et al., 2021). In another study, students showed 48.2% of an elevated perceived stress level, 37% of anxiety, and 31% of depression during the period of the COVID-19 in USA (Aiyer et al., 2020). Two-fifth of students reported PTSD symptoms, one-fourth of students reported depression, and about one-fifth of students reported anxiety and stress in Nigeria (Adewale et al., 2021). International students living far from their families have had higher psychiatric suffering that Chinese students studying in the USA had a prevalence of 49.4% anxiety, 39.8% depression, and 37.5% PTSD (Song et al., 2020).

Anxiety and depression were predicted by low income or loss of income, living place, presence of children in the home, personal characteristics, current smokers, and preexisting health conditions in self and others during the lockdown and social distancing period (Hoyt et al., 2020; Kikuchi et al., 2020; Kilani et al., 2020; Shevlin et al., 2020; Kar et al., 2021). Strong gender influence was seen in that female students had a higher prevalence of depression and anxiety (Aiyer et al., 2020). Females and last-term students had higher worries about not finding a job after graduation, from the study by Aslan (2021a) applied during the first wave of the pandemic in 2020. Moreover, female students displayed a higher fear of COVID-19 than male students during the lockdown among undergraduates from Ecuador (Rodríguez-Hidalgo et al., 2020). Age as a risk factor is noticeable among students, showing that being below the age of 24 years was linked to higher anxiety and depression (Debowska et al., 2022). Living in an urban area was linked to lower anxiety in China (Cao et al., 2020), but in Bangladesh to higher anxiety and depression (Islam et al., 2020). Students living in a village had the worst living conditions in Turkey (Aslan, 2021a). As a result, rural dwelling, being female, young, and being at risk of contact with COVID-19 were risk factors, while living in urban areas, living with parents, and having a stable family income were positive factors during the pandemic (Kar et al., 2021).

Religiosity and spirituality are fairly interconnected and difficult to separate, and they help in finding value in one’s life, peace, and a sense of connection, affecting personal and academic life (Coppola et al., 2021). People with positive religious coping, intrinsic religiosity, and trust in God with guides, norms, and beliefs as a supporting system have less stress, creating positive impacts on them.

Greater meaning-based coping, the positive reappraisal, and reinterpretation of a stressor can make people more psychologically resilient against traumatic events. Spiritual well-being linked to a greater sense of purpose, meaning in life, satisfaction with life, and lower death anxiety (Ishabiyi and Khan, 2020; Arslan and Yıldırım, 2021) is a protective method, leading to lower stress and better psychological functioning on subjective well-being through dealing with fear during the pandemic (Chang et al., 2019; Arslan and Yıldırım, 2021). Psychological and physical health can be protected through spirituality and religious practices.

Moreover, spiritual well-being functions as a protective factor against addictive or suicidal behaviors (Arslan and Yıldırım, 2021), and dying by suicide is forbidden in Islam. More older people with the inevitability of death and women because of psychological differences are involved in religious and spiritual activities more, and they feel a more significant presence of God in everyday life (Coppola et al., 2021) that God can protect people from all evil and suffering (Kowalczyk et al., 2020). However, worship services have facilitated the spread of the SARS-CoV-2 virus in some countries. Feelings of anger toward abandonment or being punished by God, doubts about the truth of one’s religious faith, questions about ultimate meaning and purpose in life, struggles with living up to one’s moral values, and increasing conflicts with other people about religion are religious struggles questioned during the pandemic (Dein et al., 2020).

## 3. Materials and methods

This research was carried out between November and December 2020 during the second wave of the COVID-19 pandemic as a semi-replication of the first wave (April–May 2020) study at different universities located in various regions of Turkey. In this study, different from the first wave study, PTSD, religiosity and spirituality, and students’ intention to die by suicide were measured. This study aimed to measure the prevalence and predictors of mental problems during the second wave of the pandemic. Furthermore, the relationships of religiosity and spirituality and physical activity with mental problems are to be determined. The following research question was put forward: What are the relationships between PTSD, anxiety, perceived stress, depression and variables, such as gender, faculty, place of residence, relationship status, level of study, PA, COVID-19 symptoms, hospitalization, death of close relatives, religiosity and spirituality level, or job loss, in students, during the second wave of the COVID-19 pandemic?

### 3.1. Assessment of socio-demographic factors

There were 754 students from Bingöl University, Bingöl (*n* = 153, 20.3%); Atatürk University, Erzurum (*n* = 265, 35.1%); Ağrıİbrahim Çeçen University, Ağrı (*n* = 142, 18.8%); and Ağrı and Iğdır University, Iğdır (*n* = 79, 10.5%)—all in the eastern part of Turkey; Bursa Uludağ University, Bursa (*n* = 27, 3.6%) and Muğla Sıtkı Koçman University, Muğla (*n* = 48, 6.4%)—all in the western part of Turkey; and Başkent University, Ankara (*n* = 40, 5.3%)—in the central part of Turkey. When choosing universities, it was taken into account that there were close friends or contacts who would assist in conducting surveys at these universities.

In 2022 in Turkey, 50.3% of university students are male, and 49.7% of university students are female (YÖK, 2022). Overall, there were 66.2% of women (*n* = 499), and 94% of them (*n* = 709) were single; 76.7% of students (*n* = 578) live in cities, and just 19.6% of them (*n* = 148) live in villages, showing that students live mainly in urban areas; 48.7% (*n* = 367) and 27.5% (*n* = 207) of students are from medical and health sciences, and social sciences, respectively. Students are mainly undergraduates, and 61.1% of them are in their third and fifth year of study. Descriptive statistics about demographics in detail can be seen in Table 1.

**TABLE 1:** Demographic characteristics of the study sample and COVID-related and psychological variables (*n* = 754).

Demographic variables	*N*	%
**Gender**		
Women	499	66.2
Men	255	33.8
**Social status**		
Married	45	6
Single	709	94
**Place of residence**		
Village	148	19.6
Town	28	3.7
City	578	76.7
**Faculty**		
Social Sciences	207	27.5
Humanistic & Art	60	8.0
Natural Sciences	21	2.8
STEM (Science, Technology, Engineering and Mathematics)	99	13.1
Medical & Health Sciences	367	48.7
**Level of study**		
Foundation (2-years programs)	233	30.9
Undergraduates	461	61.1
Master or MBA	41	5.4
Doctoral	19	2.5
**Year of study(years at university)**		
First	166	22.0
Second	247	32.8
Third	72	9.5
Fourth	183	24.3
Fifth	69	9.2
Studying more than 5 years at the university	17	2.3
**University**		
Ağrı İbrahim Çeçen University	142	18.8
Atatürk University	265	35.1
Başkent University	40	5.3
Bingöl University	153	20.3
Bursa Uludağ University	27	3.6
Iğdır University	79	10.5
Muğla Sıtkı Koçman University	48	6.4
**Exposure to COVID-19 (Yes)**		
COVID-19 symptoms of infection	222	29.4 (6.14)
Tested for the COVID-19	153	20.3 (3.35)
Hospitalization due to the COVID-19	13	1.7 (0.28)
Strict quarantine for at least 14 days	129	17.1 (5.03)
COVID-19 infection in close relatives	601	79.7 (22.06)
Death of close relatives due to the COVID-19	259	34.4 (5.31)
Job loss due to the COVID-19	330	43.8 (48.60)
Deterioration of economic status	498	66 (64.80)
**Anxiety (GAD-7)**		
Normal (0–4)	299	39.7 (12.57)
Mild (5–9)	179	23.7 (35.75)
Moderate (10–14)	117	15.5 (28.77)
Severe (15–21)	159	21.10 (22.91)
**Depression (PHQ-9)**		
Normal (0–4)	84	11.1 (9.50)
Mild (5–9)	255	33.8 (27.66)
Moderate (10–14)	137	18.2 (24.02)
Moderately severe (15–19)	143	19.0 (23.74)
Severe (20–27)	135	17.9 (15.08)
Neither depression nor anxiety diagnosis (score ≤ 10)	345	45.7 (31.0)
Anxiety diagnosis (GAD-7 ≥ 10)	276	36.6 (51.68)
Depression only diagnosis (PHQ-9 ≥ 10)	415	55.0 (62.85)
Dual anxiety and depression diagnosis (scores ≥ 10)	304	40.3 (45.54)
**PCL-C(PTSD)**		
Mild (17–29)	16	16.7 (na)
Moderate (30–44)	232	30.8 (na)
High Severity (45–85)	396	52.5 (na)
PTSD (PCL-C > 38)	468	61.2 (na)
**Satisfaction with life (SWLS)**		
Low (5–17)	410	54.4 (56.42)
Medium (18–23)	204	27.1 (24.58)
High (24–35)	140	16.6 (18.99)
**Perceived stress (PSS-10)**		
Low (0–13)	30	4 (5.59)
Medium (14–19)	89	11.8 (23.18)
High (20–40)	635	84.2 (71.23)

na, not applicable. Parentheses from previous work.

### 3.2. Procedure

A cross-national study was conducted online between 18 November and 26 December 2020, during the second wave of the COVID-19 pandemic. The survey was created *via* Google Forms. The sampling was convenience sampling, with the selection criterion being a university student. The invitation to the online study was sent to students by researchers *via* mails, WhatsApp, MsTeams, Instagram, and other social media platforms. The final total sample of university students participating in the study was 808, but 54 participants were excluded from different universities due to being low sample size; hence, it is decided to include seven university participants for comparison purposes.

### 3.3. Ethics statement

The ethics committee approved the study protocol of the University Research Committee at the University of Bingöl, Turkey, with a decision no. 92342550/044/6137. The study followed the ethical requirements about the anonymity and voluntariness of participation. Each person answered the informed consent question. Following the Helsinki Declaration, written informed consent was obtained from each student before inclusion. This study was part of an international research project: Well-being of undergraduates during the COVID-19 pandemic: International study, registered at the Center for Open Science (OSF) (Rogowska et al., 2020).

### 3.4. Measures

The Perceived Stress Scale (PSS-10) (Cohen et al., 1983) was conducted to measure whether the respondents considered the situation in their life as stressful. The PPS-10 consists of 10 items referring to the frequency of stressful events in the month preceding the study, which is assessed on a 5-point scale (0 = never to 4 = very often). PSS-10 has a Cronbach’s alpha value of 0.665.

The 7-item Generalized Anxiety Disorder (GAD-7) Scale (Spitzer et al., 2006) is a self-reported measure designed to screen for symptoms. Students rate how often they experienced anxiety symptoms in the 2 weeks preceding the study on a 4-point Likert scale (0 = not at all, 1 = several days, 2 = more than half the days, and 3 = nearly every day). The GAD-7 ranges from 0 to 21 and evaluates the minimal, mild, moderate, and severe anxiety levels, respectively, with 0–4, 5–9, 10–14, and 15–21 scores (Spitzer et al., 2006). Scores above 10 points indicate an anxiety disorder (Lee et al., 2016). The anxiety scale has a Cronbach’s alpha value of 0.912.

The Patient Health Questionnaire (PHQ-9) was used to measure depression symptoms. The PHQ-9 consists of 9 items conforming with DSM-V diagnostic criteria (APA [American Psychiatric Association], 2013). Participants used a Likert-type response scale (0 = not at all to 3 = nearly every day). The ranges of PHQ-9 scores are 0–4, normal; 5–9, mild major depressive disorder; 10–14, moderate; 15–19, moderately severe; and 20–27, severe. A cut-off score of 10 or above is recommended to screen for major depressive disorder (Kroenke et al., 2009; Praharso et al., 2017), and depression has a Cronbach’s alpha value of 0.904.

Posttraumatic stress disorder using Checklist-Civilian Version (PCL-C) (0 = never to 4 = very often), which is a 17-item self-report developed by Weathers et al. (2013), was used. The total scores range from 17 to 85, and scores of 38 or higher indicate the presence of PTSD. PCL-C has a Cronbach’s alpha value of 0.948.

The Satisfaction with Life Scale (SWLS) consists of five items using a seven-point Likert scale (from 1 = strongly disagree to 7 = strongly agree), and the scale has a Cronbach’s alpha value of 0.863.

Demographic questions are related to age, gender, place of residence, the current level of study, year of study, and types of faculty. An exposure to COVID-19 based on 8 questions about the coronavirus’ consequences (1 = yes, 0 = no), the perceived impact of coronavirus (PIC) on the students’ lives using 5 statements (from 1 = strongly disagree, to 5 = definitely agree), physical activity during a week physical exercise (from 0 = not 1 day to 7 = 7 days a week), and physical exercise minutes per week are other parts of the survey.

The scales used in this study have been translated from English to Turkish by using previous studies. The reliability and validity of PSS-10 (Erci, 2009), GAD-7 (Konkan et al., 2013), PHQ-9 (Sari et al., 2016), and PCL-C (Kocabaşoğlu et al., 2005) scales were checked for preventing misunderstandings and for overcoming language barriers in Turkey. SWLS Scale was translated from English to Turkish by the authors. Cronbach’s alpha is used to measure reliability scales, and George and Mallery (2003) suggested Cronbach’s alpha ≥ 0.6 as acceptable. SWLS, PSS-10, PCL-C, anxiety, and depression scales met the reliability criteria.

### 3.5. Statistical analysis and sampling

A preliminary analysis of the prevalence of all variables was examined before statistical tests were applied. A one-way ANOVA was performed to test the differences in mean scores. The normality assumption was checked using skewness and kurtosis scores and their decision rules: skewness and kurtosis values < | 1| = acceptable for normality (George and Mallery, 2016). Next, the multinomial logistic regression analysis was performed to test the odds ratio (OR) with 95% CI. All analyses were performed using SPSS 22.0 version.

The minimum required sample size at 99% CI and 5% margin of error and with the information from the previous study p (proportion of students with psychological problems) = 0.6 is 640 through the Cochran formula for an unlimited population size, and our sample size (*n* = 754) met the minimum sample size criteria. The analysis encompassed descriptive statistics: mean (M), standard deviation (SD), and 95% of confidence interval (CI) with lower limit (LL) and upper limit (UL).

## 4. Results

### 4.1. Descriptive statistics and the prevalence of perceived stress, anxiety, depression, and PTSD among university students

High perceived stress, moderate generalized anxiety disorder (GAD), mild depression symptoms (PHQ), high severity of PTSD, and moderate satisfaction were found among students as shown in Table 2. The greatest increase was seen in anxiety from the mean of 6.43 (April–May 2020) to 9.66 (December–November 2020) as the pandemic was getting longer; students become more anxious while there were no considerable changes in perceived stress, depression, and satisfaction with life. A study by Weathers et al. (2013) showed that a score of 38 or higher indicates the presence of PTSD. In our study, the average total score of PTSD was 47.39, which shows that the pandemic has long-term effects on the mental well-being of students. Job search and professional development, relationships with colleagues and friends, financial situation, and completion of the semester and graduation were the highest rising negative impacts of the COVID-19 pandemic associated with the fear of the situation from the perception of the impact of COVID-19 on well-being as shown in Table 2 compared to the first wave. Relationships with loved ones, family, and friends have worsened as the most substantial change in the perception of the COVID-19 impact on the well-being group (PIC). It can be stated that the negative impacts of the pandemic have increased with a grand mean of 3.79 when compared to April 2020 with a grand mean of 3.75. Sufficient physical activity (PA) > 150 min weekly and insufficient PA < 150 min weekly are the categorization of physical activity according to WHO recommendations. Students were 75 min active weekly in April 2020, while they were 22.13 min active weekly during the second wave of the pandemic with insufficient physical activity (PA < 150 min weekly); the students’ physical inactivity during the second wave could be explained mainly by restrictions and lockdowns.

**TABLE 2:** Descriptive statistics (*n* = 754).

				95% CI	
Variable	Range	M	SD	LL	UL
Perceived stress	4–40	24.32 (22.74)	5.74	23.91	24.73
PCL-C (PTSD)	5-85	47.39 (na)	17.69	46.13	48.66
Anxiety	0–21	9.66 (6.43)	5.40	9.28	10.05
Depression	0–27	12.24 (12.42)	6.92	11.74	12.73
Satisfaction with life	5–35	16.79 (16.72)	6.94	16.30	17.29
Perception of the impact of COVID-19 on well-being	5–25	18.95 (18.74)	4.57	18.62	19.28
Completion of the semester and graduation	1–5	3.73 (3.36)	1.40	3.63	3.83
Job search and professional development	1–5	3.98 (3.67)	1.28	3.89	4.07
Financial situation	1–5	3.75 (3.82)	1.32	3.66	3.85
Relationships with loved ones: family	1–5	3.64 (2.44)	1.30	3.55	3.74
Relationships with colleagues: friends	1–5	3.83 (2.57)	1.21	3.74	3.91
Physical activity per week (days)	0–7	1.72 (na)	1.98	1.57	1.86
Physical activity during pandemic (minutes per week)	–	22.13 (75.53)	28.40	20.10	24.16
How religious do you consider yourself to be?	0-3	1.9483 (na)	0.71	1.89	1.99
How spiritual do you consider yourself to be?	1-4	2.96 (na)	0.71	2.91	3.01

M, mean; SD, standard deviation; CL, confidence interval; LL, lower limit of the confidence interval; UL, upper limit of the confidence interval; na, not applicable; parentheses from previous work.

COVID-19 infection in close relatives (79.7%), declining economic status (65%), and losing a job by a student or in the student’s family (43.8%) were the strongest results of exposure to COVID-19. During the second wave, exposure to COVID-19 increased dramatically, especially in the death rates of relatives, as shown in Table 1. In contrast, there was not a noteworthy change in economic status. Even though 29.4% of them have shown symptoms of COVID-19 infection, just 17.1% of them had strict quarantine for at least 14 days, and 13 students were hospitalized due to COVID-19 infection; 34.4% (259) of them have stated the COVID-19-related deaths among their families.

Only 4% of students were characterized with low stress, while 84.2% showed a high stress level with a 13% increase compared to April 2020. More than half of the students had low satisfaction, similar to the first wave period; 15.5 and 21.10% of students have moderate and severe GAD symptoms, representing 36.6% anxiety diagnosis (GAD-7 ≥ 10), showing an improvement in GAD management compared to 51.68% of the first wave period; 17.9% of students have severe depression symptoms, higher than the 15.08% of April 2020 prevalence, while moderate and moderately severe symptoms decreased, but still 55.0% depression prevalence was high; 45.7% of students do not show any symptoms of depression and anxiety diagnosis (score ≤ 10), whereas 40.3% of them presented dual anxiety and depression diagnosis (score ≥ 10). About half of students from the PHQ-9 depression symptoms scale: “*Thoughts that you would be better off dead, or thoughts of hurting yourself in some way?*” item had thoughts of suicide with several days (26.9%), more than half the days (9.7%), and nearly every day (10.6%), and 20.3% of students were under considerable suicide risk. Over half of the students’ population (61.2%) have presented clinical symptoms of PTSD (PCL-C > 38), and 30.8% of them have shown moderate PTSD. The results in detail are presented in Tables 1, 2.

### 4.2. Correlation among variables

There is a significant negative correlation between satisfaction with life and psychological problems (anxiety, depression, and PTSD) except stress. Moreover, SWLS has a significant positive correlation with religiosity and spirituality level; religious and spiritual students can be more satisfied, and they have less psychological problems with significant negative correlations. The correlation between perceived stress (PSS) and COVID-19 impact (PCI) is positive and significant on students’ well-being (*r* = 0.269; *p* < 0.001). Furthermore, there are significant positive correlations between perceived stress and anxiety, depression and PTSD. Generalized anxiety disorder intensity had a high correlation with depression (*r* = 0.799; *p* < 0.01) and PTSD (*r* = 0.650; *p* < 0.01) with a large effect size based on Turney’s (2022) coefficient of determination (*r*^2^) calculation that anxiety can be turned into more dangerous mental problems. Physical activities are inversely correlated with depression (*r* = −0.117; *p* < 0.05) and PTSD (*r* = −0.093; *p* < 0.05) but positively correlated with satisfaction with life (*r* = 0.079*; *p* < 0.05). Pearson’s *r* coefficients are presented in Table 3.

**TABLE 3:** Correlation matrix with Pearson’s *r* coefficient (*n* = 754).

Variable	SWL	Anxiety	PTSD	Stress	Depression	Impacts	Religiosity	Spirituality	PA
**SWL**	1	-0.234**	-0.256**	0.001	-0.285**	-0.066	0.133**	0.123**	0.079*
**Anxiety**	-0.234**	1	0.650**	0.354**	0.799**	0.215**	-0.136**	-0.122**	-0.063
**PTSD**	-0.256**	0.650**	1	0.377**	0.690**	0.269**	-0.127**	-0.055	-0.093*
**Stress**	0.001	0.354**	0.377**	1	0.308**	0.162**	0.041	0.046	-0.002
**Depression**	-0.285**	0.799**	0.690**	0.308**	1	0.172**	-0.184**	-0.114**	-0.117**
**Impacts**	-0.066	0.215**	0.269**	0.162**	0.172**	1	-0.019	-0.008	-0.054
**Religious**	0.133**	-0.136**	-0.127**	0.041	-0.184**	-0.019	1	0.420**	0.018
**Spiritual**	0.123**	-0.122**	-0.055	0.046	-0.114**	-0.008	0.420**	1	-0.010
**PA**	0.079*	-0.063	-0.093*	-0.002	-0.117**	-0.054	0.018	0.010	1

*p < 0.05; **p < 0.01.

### 4.3. Significance of differences according to demographic variables and exposure to COVID-19

One-way ANOVA was applied to find the significance of differences in satisfaction with life, perceived stress, anxiety, depression, and PTSD scales based on categorical ranges as shown in Table 1 and normality, assuming that each group has equal variance. Significant differences according to gender, social status, place of residence, level of study, universities, year of study, faculty, and exposure to COVID-19 variables were searched using a one-way ANOVA test (*p* < 0.05 is considered significant difference), and a *post-hoc* Tukey’s multiple comparison test was applied for pair comparisons shown in Table 4. There were no significant differences in relation to university types, gender, COVID-19 symptoms, strict quarantine, and COVID-19 infection (*p* > 0.05) for life satisfaction. However, there are significant differences according to social status (*F* = 13.7; *p* = 0.00) in favor of married students (μ = 20.4), with medium satisfaction, while single students (μ = 16.5) had low satisfaction. Students living in villages had the worst life satisfaction, and significant differences were found in favor of students living in cities (city and big city) compared to villages by *post hoc* analysis. However, all students had low satisfaction (5–17), while just students living in big cities have medium satisfaction (μ = 17.8). Master and Ph.D. students had medium satisfaction, while students at other levels had low satisfaction with significant differences in the level of study and year of study (*p* < 0.05). Students in their first and second years had the lowest satisfaction. Being hospitalized (μ = 23.6) has significant and high satisfaction than not hospitalized students with low satisfaction (μ = 16.6), and students tested for COVID-19 had medium satisfaction than non-tested students with low satisfaction. Deaths of close relatives, job losses, and deterioration of economic status were other significant reasons for the low satisfaction with significant differences. Place of residence, universities, and year of study were not significantly different for the stress scale (*p* > 0.05). Female and single students had higher stress with significant differences (*p* < 0.05). Master and Ph.D. students had lower stress significantly different (*p* < 0.05) from 2 years and undergraduate students. Having COVID-19 infection, deaths of a relative, losing a job, and worsening economic situation with significant differences are sources of higher stress than students selected (No) in exposures to COVID-19 items. Female students (μ = 10.2) with moderate anxiety than male students (μ = 8.52) with mild anxiety, students in big cities (μ = 10.1) and villages (μ = 10.0) with moderate anxiety than other residence places having mild anxiety, both Ağrı and Muğla Sıtkı Koçman Universities with moderate anxiety than other universities with mild anxiety, third-year students (μ = 12.1) having significant differences from students studying in other years with mild anxiety symptoms, having symptoms of coronavirus, deaths of a relative, losing a job, and worsening economic situation were variables having significant differences in the anxiety scale. In contrast, hospitalization, tested for COVID-19, strict quarantine, and having COVID-19 infection variables had no significant differences in the anxiety scale.

**TABLE 4:** Significant differences according to demographic variables and exposure to COVID-19 items.

Variable		SWL			Stress			Anxiety			Depression			PTSDs		
		**Mean**	***F***	**Sig.**	**Mean**	***F***	**Sig.**	**Mean**	***F***	**Sig.**	**Mean**	***F***	**Sig.**	**Mean**	***F***	**Sig.**
Gender	Female (*n* = 499)	16.8	0.04	0.82	24.9	16.6	0.000*	10.2	17.4	0.000*	12.8	13.3	0.000*	49.7	27.9	0.000*
	Male (*n* = 255)	16.7			23.1			8.52			10.9			42.7		
Social status	Married (*n* = 45)	20.4	13.7	0.00*	22.2	6.585	0.010*	7.7	6.170	0.013*	9.0	10.321	0.001*	41.2	5.823	0.016*
	Single (*n* = 709)	16.5			24.4			9.7			12.4			47.7		
Place of residence	Village (*n* = 148)	14.7	6.9	0.00*	23.9	0.81	0.48	10.0	2.6	0.040*	12.2	1.7	0.16	47.7	2.0	0.11
	Town (*n* = 28)	15.9			23.8			8.3			11.5			47.5		
	City (*n* = 291)	16.8			24.1			9.1			11.6			45.5		
	Big City (*n* = 287)	17.8			24.7			10.1			12.9			49.0		
Level of study	Foundation (*n* = 233)	16.1	3.50	0.02*	24.3	5.6 1 and 3 2 and 3	0.004*	9.4	5.26	0.005*	12.0	4.71	0.009*	47.2	2.11	0.122
	Undergraduates (*n* = 461)	16.8			24.6			10.0			12.6			48.0		
	Master and PhD (*n* = 60)	18.8			21.9			7.7			9.7			43.0		
Universities	Ağrı (*n* = 142)	15.7	1.8	0.08	23.9	0.44	0.85	10.5	1.9	0.006*	14.0	3.7	0.001*	51.2	2.42	0.025*
	Atatürk (*n* = 265)	17.1			24.4			9.3			11.8			46.7		
	Başkent (*n* = 40)	19.6			24.9			9.2			12.2			45.7		
	Bingöl (*n* = 153)	16.6			24.1			9.2			11.8			46.9		
	Bursa (*n* = 27)	16.3			25.2			12.1			14.6			50.5		
	Iğdır (*n* = 79)	16.5			24.7			9.2			10.0			42.5		
	Muğla (*n* = 48)	16.4			23.8			10.0			12.1			48.54		
Year of study	First (*n* = 166)	16.6	2.5	0.03*	23.6	0.70	0.59	8.4	6.2	0.00*	10.8	5.0	0.001*	43.7	4.2	0.002*
	Second (*n* = 247)	15.8			24.4			9.7			12.2			48.0		
	Third (*n* = 72)	17.6			24.4			12.1			15.1			53.4		
	Fourth (*n* = 183)	17.2			24.4			9.5			12.2			46.7		
	Fifth and over (*n* = 86)	18.1			24.7			9.9			12.3			48.8		
COVID-19 symptoms	No (*n* = 532)	16.9	0.77	0.37	24.0	3.2	0.070	9.2	10.5	0.001*	11.7	9.0	0.003*	45.6	17.0	0.000*
	Yes (222)	16.4			24.9			10.6			13.4			51.4		
Tested for the COVID-19	No (*n* = 601)	16.5	4.1	0.04*	24.1	2.9	0.08	9.4	3.3	0.068	11.9	5.8	0.015*	47.0	0.929	0.335
	Yes (*n* = 153)	17.8			25.0			10.3			13.4			48.6		
Hospitalization	No (*n* = 741)	16.6	12.9	0.00*	24.3	0.51	0.472	9.6	0.40	0.52	12.2	0.001	0.972	47.40	0.017	0.897
	Yes (*n* = 13)	23.6			25.4			10.6			12.3			46.7		
Strict quarantine	No (*n* = 625)	16.8	0.28	0.59	24.1	3.1	0.07	9.6	0.43	0.50	12.1	0.56	0.444	47.1	0.66	0.41
	Yes (*n* = 129)	16.5			25.1			9.95			12.66			48.5		
COVID-19 infection	No (*n* = 153)	17.5	2.08	0.149	23.4	4.6	0.031*	8.96	3.289	0.070	11.0	6.14	0.013*	44.15	6.482	0.011*
	Yes (*n* = 601)	16.6			24.5			9.8			12.5			48.22		
Death of close relatives	No (*n* = 495)	17.1	3.9	0.04*	23.6	17.51	0.000*	9.1	15.43	0.00*	11.5	15.43	0.000*	45.8	10.7	0.001*
	Yes (*n* = 259)	16.1			25.5			10.7			13.5			50.2		
Job loss	No (*n* = 424)	17.8	24.7	0.00*	23.8	7.9	0.005*	8.8	24.22	0.000*	11.1	25.10	0.000*	44.	37.1	0.00*
	Yes (*n* = 330)	15.3			24.9			10.7			13.6			51.7		
Deterioration of economic status	No (*n* = 256)	18.4	21.7	0.00*	23.1	16.8	0.00*	8.6	15.12	0.000*	10.6	21.5	0.000*	41.62	43.57	0.00*
	Yes (*n* = 498)	15.9			24.9			10.2			13.0			50.36		

*The mean difference is significant at the 0.05 level. Mean, mean of summed scale.

Being female (μ = 12.8) and single (μ = 12.4) with moderate depression, having 2 years or 4 years of studies, being a third-year student with moderate depression, and being students of Ağrı University (μ = 14.0) and Bursa Uludağ University (μ = 14.6) with moderately severe depression were measured with significant differences in the depression scale. The exposures to COVID-19 have increased the depression rate in the second wave, and losing jobs and worsening economic situations were the most explicit source of depression symptoms. Female students (μ = 49.7) were more inclined to PTSD than male students (μ = 42.7) having moderate PTSD symptoms. Single students with high PTSD score were more inclined to PTSD. Students from Iğdır University had moderate PTSD symptoms, while students from other universities, especially Ağrıİbrahim Çeçen University students (μ = 51.2), had high PTSD symptoms. Third-year students showed the highest PTSD symptoms, having significant differences from first- and fourth-year students. The COVID-19 symptoms and infections, deaths of relatives, and worsening economic situation could be the reasons for higher significant PTSD.

### 4.4. Predictors of stress, anxiety, depression, and PTSD

A logistic regression prediction model exploring whether socio-demographic variables (gender, place of residence, level of study, religion, exposure to COVID-19, etc.) are predictors of PTSD, anxiety, depression, and perceived stress among students during the COVID-19 pandemic was developed.

A likelihood ratio test and Nagelkerke’s pseudo *R*-square values were applied to test the validity of the model, and stress (*R*-square: 0.252; χ^2^ = 134.9, *p* = 0.000 < 0.05), anxiety (*R*-square: 0.264; χ^2^ = 212.21, *p* = 0.000), depression (*R*-square: 0.261; χ^2^ = 208.22, *p* = 0.000), and PTSD (*R*-square: 0.258; χ^2^ = 190.06, *p* = 0.000) have fitted model values. Religious level (95%, χ^2^ = 8.492, *p* = 0.014), gender (95%, χ^2^ = 18.99, *p* = 0.000), and losing jobs (95%, χ^2^ = 14.22, *p* = 0.001) were predictors of stress. Relationship status (95%, χ^2^ = 12.34, *p* = 0.06), year of study (95%, χ^2^ = 43.32, *p* = 0.000), PA (95%, χ^2^ = 7.72, *p* = 0.05), symptoms of COVID-19 (95%, χ^2^ = 14.43, *p* = 0.002), death of close relatives (95%, χ^2^ = 9.8, *p* = 0.020), and job loss (95%, χ^2^ = 9.29, *p* = 0.026) were significant predictors of anxiety; religious level (95%, χ^2^ = 10.27, *p* = 0.016), relationship status (95%, χ^2^ = 11.47, *p* = 0.009), year of study (95%, χ^2^ = 23.97, *p* = 0.02), PA (95%, χ^2^ = 9.29, *p* = 0.026), death of close relatives (95%, χ^2^ = 11.54, *p* = 0.009), job loss (95%, χ^2^ = 9.26, *p* = 0.026), and deterioration of economic status (95%, χ^2^ = 7.76, *p* = 0.026) were significant predictors of depression; and religious level (95%, χ^2^ = 7.2, *p* = 0.027), gender (95%, χ^2^ = 17.6, *p* = 0.000), year of study (95%, χ^2^ = 26.07, *p* = 0.001), university (95%, χ^2^ = 11.6, *p* = 0.003), PA (95%, χ^2^ = 11.6, *p* = 0.003), symptoms of COVID-19 (95%, χ^2^ = 11.69, *p* = 0.003), job loss (95%, χ^2^ = 10.41, *p* = 0.005), and deterioration of economic status (95%, χ^2^ = 20.174, *p* = 0.000 < 0.05) were significant predictors of the PTSD as presented in Table 5.

**TABLE 5:** Predictors of models from a socio-interpersonal perspective by multinomial logistic regression.

Variable	Stress		Anxiety		Depression		PTSD	
	**Chi-square (χ^2^)**	**Sig.**	**Chi-square (χ^2^)**	**Sig.**	**Chi-square (χ^2^)**	**Sig.**	**Chi-square (χ^2^)**	**Sig.**
Religiosity level	8.492*	0.014	3.802	0.284	10.275*	0.016	7.250*	0.027
Spirituality level	2.921	0.232	6.828	0.078	1.821	0.610	1.965	0.374
Gender	18.99*	0.000	5.499	0.139	6.335	0.096	17.604*	0.000
Relationship status	4.284	0.117	12.343*	0.006	11.472*	0.009	5.048	0.080
Place of residence	9.569	0.144	14.574	0.103	9.236	0.416	11.483	0.075
Faculty	4.706	0.789	14.334	0.280	14.708	0.258	5.042	0.753
Level of study	4.184	0.382	11.457	0.075	5.187	0.520	1.575	0.813
Year of study	13.390	0.099	43.320*	0.000	23.979*	0.020	26.071*	0.001
University	18.197	0.110	20.138	0.325	25.367	0.115	21.304*	0.046
PA	0.332	0.847	7.721*	0.05	9.682*	0.021	11.645*	0.003
COVID-19 symptoms	0.255	0.880	14.439*	0.002	6.164	0.104	11.692*	0.003
Tested for the COVID-19	1.093	0.579	2.134	0.545	3.888	0.274	0.857	0.651
Hospitalization	2.855	0.240	2.677	0.444	2.436	0.487	0.515	0.773
Strict quarantine	2.801	0.247	2.793	0.425	3.932	0.269	2.101	0.350
COVID-19 infection	1.527	0.466	5.132	0.162	1.759	0.624	0.283	0.868
Death of close relatives	3.204	0.201	9.829*	0.020	11.543*	0.009	4.059	0.131
Job loss	14.222*	0.001	9.291*	0.026	9.261*	0.026	10.415*	0.005
Deterioration of economic status	2.569	0.277	7.394	0.060	7.762*	0.05	20.174*	0.000

PA, physical activity. *p < 0.05.

The last category was taken as a baseline for both dependent and independent variables. Odds ratio tests with significant (*p* < 0.05) values are analyzed in each model for stress, anxiety, depression, and PTSD. Religious and spirituality levels were entered in the model as covariates, while other independent variables were entered under the factor section in multinomial logistic regression. Demographic characteristics are shown in Table 1, and PA (<150 min per week = 0; >150 min per week = 1) and exposure to COVID-19 as yes or no were sub-categories of the variables.

The multinomial logistic estimates compare female students to male students for medium stress relative to high stress given the other variables in the model are held constant [coefficient (β) = −1.134, odds ratio (OR) (Exp (B-odds ratio) = 0.322; χ^2^ (1) = 18.720, *p* = 0.000]; the multinomial logistic estimate score is 1.134 unit lower for medium stress relative to high stress, and male students are more likely to have higher stress. The probability of female students having low stress with respect to high stress was 0.322 times lower than male students. Religious students show 0.863 unit lower for low stress relative to high stress [β = −0.863, OR = 0.422; χ^2^ (1) = 8.49, *p* = 0.004]. The married students for medium stress relative to high stress were 2.56 times higher than single students [β = 0.941, OR = 2.56; χ^2^ (1) = 4.31, *p* = 0.038]. Atatürk University [β = −1.378, OR = 0.252; χ^2^ (1) = 4.38, *p* = 0.036] and Iğdır University [β = −1.97, OR = 0.138; χ^2^ (1) = 5.18, *p* = 0.023] students for medium stress relative to high stress showed 1.37 and 1.97 lower than students in Muğla University. Students and their families not losing their jobs were more likely to have medium stress relative to high stress than students losing their jobs, 2.87 times higher relative probability [β = 1.056, OR = 2.87; χ^2^ (1) = 12.95, *p* = 0.000]. Participants exposed to COVID-19 were between 1 and 3.5 times more likely to report high stress (OR = 1.08–3.47).

Female students for normal anxiety (0–4) relative to severe anxiety (15–21) showed lower anxiety than male students [β = −0.6, OR = 0.549; χ^2^ (1) = 3.99, *p* = 0.046]. The relative risk of having normal anxiety would be 0.549 times more likely when the other variables in the model are held constant. Undergraduate students had lower anxiety for normal anxiety relative to severe anxiety [β = −1.69, OR = 0.183; χ^2^ (1) = 6.39, *p* = 0.011] and for mild anxiety relative to severe anxiety [β = −1.709, OR = 0.181; χ^2^ (1) = 8.06, *p* = 0.005] than master and Ph.D. students. First-year students for normal anxiety relative to severe anxiety [β = 1.95, OR = 7.06; χ^2^ (1) = 10.52, *p* = 0.001] had higher anxiety, while third-year students for mild anxiety relative to severe anxiety [β = −1.024, OR = 0.359; χ^2^ (1) = 4.52, *p* = 0.033] had lower anxiety than sixth-year students. Furthermore, spiritual students showed 1.44 times higher anxiety for mild anxiety relative to severe anxiety [β = 0.371, OR = 1.44; χ^2^ (1) = 5.27, *p* = 0.022]. Participants having COVID-19 symptoms [β = 0.93, OR = 2.54; χ^2^ (1) = 9.6, *p* = 0.002] and losing jobs [β = 0.8, OR = 2.27; χ^2^ (1) = 5.1, *p* = 0.023] were 2.27 times and 2.54 more likely to report high anxiety in relation to normal anxiety, respectively. Hospitalization with OR = 4.99 (*p* < 0.05) had the highest relative risk for severe anxiety than normal anxiety.

Being religious (β = −0.397) and spiritual (β = −0.321) and involving physical activities (β = −0.011) represent effective coping factors against depression among university students (negative coefficients being observed). Women showed lower depression than men for normal (0–4) depression relative to severe (20–27) depression (β = −0.758, OR = 0.469; χ^2^ (1) = 5.03, *p* = 0.025 < 0.05). First-year students compared to 5 + level students had 3.65 times more depression for normal depression relative to severe depression [β = 1.29, OR = 3.65; χ^2^ (1) = 4.004, *p* = 0.045]. Being religious was found to be a slightly higher risk for students [β = 0.548, OR = 1.72; χ^2^ (1) = 9.65, *p* = 0.002] for mild in relation to severe depression. Participants exposed to COVID-19 were between 1.09 and 2.7 times more likely to report high depression. Job loss with OR = 2.7 and economic deterioration with OR = 2.19 are significant factors (*p* < 0.05) behind severe depression as shown in Table 5.

Female students for low to high severity PTSD [β = −0.993, OR = 0.371; χ^2^ (1) = 16.266, *p* = 0.000] and for medium to high severity PTSD [β = −0.494, OR = 0.61; χ^2^ (1) = 6.26, *p* = 0.012] showed less likely than men. First-year students are 4.5 times more likely to have severe PTSD than 5 + students [β = 1.508, OR = 4.5; χ^2^ (1) = 9.37, *p* = 0.002]. Religious students had higher PTSD scores from medium to high severity [β = 0.348, OR = 1.41; χ^2^ (1) = 5.9, *p* = 0.014]. Religious students showed lower PTSD [β = −0.275, OR = 0.759; χ^2^ (1) = 4.01, *p* = 0.045] in the overall model, while students being spiritual have a coefficient value of −0.162 but not significant (*p* > 0.05). Participants not exposed to COVID-19 were in general less likely to report severe PTSD symptoms but all items were found not significant (*p* > 0.05). Having COVID-19 symptoms (OR = 1.4), job losses (OR = 1.36), and economical determination (OR = 1.30) have higher relative risks of PTSD. PA for severe PTSD had a significant negative coefficient (β = −007, OR = 0.99; Wald = 5.21, *p* = 0.023).

## 5. Discussion

Overall, 84.2% high perceived stress (20–40), 36.6% anxiety diagnosis (GAD-7 ≥ 10), 55.0% depression diagnosis (PHQ-9 ≥ 10), and 61.2% PTSD (PCL-C > 38) shown in Table 1 were the prevalence of psychological factors. Anxiety and depression rates decreased compared to the first wave of the pandemic. In contrast, stress increased during the second wave. More than half of students (52.5%) showed high-severity PTSD, and the pandemic has long-term effects on students’ well-being. The students’ prevalence from different studies before the pandemic in Turkey was 17–23% depression and 35% anxiety in the study by Deniz and Sümer (2010); 29.5% depression, 50.3% anxiety, and 39.9% stress in the study by Baykan et al. (2012); and stress level: 55.4% normal, 19.2% mild, 24% moderate, 0.6% advanced, and 0.9% very advanced; anxiety: 47% normal, 6% mild, 24.3% moderate, 13.8% advanced, and 5.4% very advanced; and depression: 65.3% normal, 16.2% mild, 11.1% moderate, 4.5% advanced, and 3% very advanced (Üstün and Bayar, 2015), indicating a high increase in the prevalence of psychological problems compared to our results. As the weeks progress, stress, anxiety, and depression levels have increased, while the quality of life has decreased in the study by Jojoa et al. (2021). Overall, 64.6% depression, 48.6% anxiety, 45.2% stress, and 34.5% PTSD from May 11 to 15, 2020, showed that PTSD has increased in the second wave (Cam et al., 2021), supporting mainly our study. High PSS (84.2%), severe anxiety (21.10%), and severe depression (17.9%) prevalence were risk factors requiring precautions. In this study, the highest increase was seen in anxiety and perceived stress, while there was no considerable change in depression. However, 17.9% of students have severe depression symptoms, higher than the 15.08% of April 2020 prevalence, while moderate and moderately severe symptoms decreased. There are more depression cases among students in Turkey compared to 36% pooled depressive symptoms and 32% anxiety symptoms prevalence around the world (Deng et al., 2021). Dilmen Bayar et al. (2021) found medium-level stress and depression in August–December 2020, and perceived stress is still a problem, while there was an improvement in depression. High perceived stress, moderate generalized anxiety disorder (GAD), mild depression symptoms (PHQ), high severity PTSD, and moderate satisfaction were found in the second wave, and the greatest increase was seen in anxiety, showing that the effects of COVID-19 have led to profound risks on mental well-being with high severity of PTSD (61.2%).

Even though there are decreases in the prevalence of dual anxiety and depression diagnosis (40.3%) and depression-only diagnosis (55.0%) (scores ≥ 10) compared to Aslan et al. (2020) study, the rates are still high. Deng et al. (2021) found 61% of students having depressive symptoms and 49% of students having anxiety symptoms with financial difficulties, stating that financial problems have strong effects on mental well-being. Twenty-seven studies reported a pooled prevalence of 23, 13, and 8% for mild, moderate, and severe depressive symptoms, respectively (Deng et al., 2021), and moderate and severe depressive symptoms are higher in Turkey. A pooled depressive symptoms prevalence of students: 24% from Chinese students, 70% from Bangladeshi students, 55% from American students, and 29% from French students; and a pooled anxiety symptoms prevalence: 23% from Chinese students, 73% from Bangladeshi students, 74% from American students, 42% from French students, and 56% from Spanish students were found by Deng et al. (2021). The prevalence of PTSD was 53.8% during the outbreak period in China (Wang et al., 2020). Turkey students generally showed a lower anxiety rate. However, students in Turkey showed higher depression (55.00%) than the Chinese and French students and better scores than Bangladeshi, American, and Spanish students. A high PTSD rate of 61.2% implies that students need mental support in Turkey. Worsened and volatile economy with financial difficulties and insufficient government support could be reasons for that high rate. Furthermore, increased effects: deaths of close relatives due to COVID-19 (from 5.31 to 34.4%), tested for coronavirus (from 3.35 to 20.3%), strict quarantine for at least 14 days (from 5.03 to 17.1%) of the pandemic, and increased unemployment have negatively influenced students well-being. The worsened relationships of students with families and friends and decreased physical activities (Aslan, 2021b) could be other reasons for mental problems in Turkey.

Younger students (aged 18–20 years), single, and females were more vulnerable to a traumatic event (Chhetri et al., 2021; Chodkiewicz et al., 2021). Cantürk (2014) explained higher significant stress, anxiety, and depressions level before the pandemic with hormonal changes and expression of emotions and thoughts regarding their social situation. Twenty-two studies reported subgroup data by gender for anxiety symptoms, with a pooled prevalence of 44% for female students and 37% for male students (Deng et al., 2021). Women were more likely to report feeling of more stress than men in the second wave (Hutcheson et al., 2021). Indeed, it has been found that the likeliness of developing PTSD following exposure to a traumatic case is two times that for women than for men (Cam et al., 2021). In this study, younger students, females, and single students had higher stress with significant differences. Female students showed moderate anxiety and depression and high-severity PTSD with higher significant mean differences as shown in Table 4, more inclined to mental problems than male students. Students in their first and second years had the lowest satisfaction. First-year students were 4.5 times more likely to have severe PTSD than 5 + students. Third-year students with moderate depression showed the highest PTSD symptoms. Married students had better satisfaction, while students living in villages had the worst satisfaction in life. Perceived stress is strongly related to anxiety and depression symptoms (Salleh, 2008; Mills et al., 2014; Aslan et al., 2020; Dilmen Bayar et al., 2021). In this study, there was a significant positive correlation between perceived stress and anxiety, depression, and PTSD, and high continuous stress can be explained by high perception of COVID-19 impacts. As people are satisfied more with life, they are less inclined to mental problems. Physical activities and turning back to normal life routines can decrease stress. Religious students show 0.863 unit lower for low stress relative to high stress. Being religious and spiritual and having physical activities have negative coefficients for severe depression to normal depression. Furthermore, religious students showed lower PTSD. Ağrıİbrahim Çeçen University and Muğla Sıtkı Koçman University students showed higher anxiety than other universities. Ağrıİbrahim Çeçen University and Bursa Uludağ University students had faced moderately severe depression. Students from Iğdır University had moderate PTSD, while students from other universities, particularly from Ağrıİbrahim Çeçen University students, had high PTSD symptoms, showing that Ağrıİbrahim Çeçen University students were under higher long-term mental problems compared to other universities. The fact that some universities (Ağrıİbrahim Çeçen University, Bingöl University, and Iğdır University) were established in 2007–2008 and not yet institutionalized may have contributed to these differences. In addition, political groupings and interest seeking in some universities may have had negative results on students. Therefore, individual studies for each university may be necessary to explain these significant differences.

Psychiatric sufferings can cause suicides. Correlations with suicidal attempts were found with mental disorders in the past. Some people died by suicide because of being infected and the economic crisis created by the COVID-19 pandemic (Jawad et al., 2020); 24% of the respondents reported having experienced suicidal thoughts compared to 10% of adult participants who had suffered from suicidal thoughts in Poland during the first wave of the pandemic, and prolonged pandemic increased the intentions of suicides (Chodkiewicz et al., 2021); 10.7% of respondents seriously considering suicide in the last 30 days were found, and this rate was 25.5% among young people (aged 18–24 years) in the USA in June 2020 (Czeisler et al., 2020). Anxiety and depressive disorders are associated with suicidal thoughts and low educational performances (ignoring classes, low grades, not submitting homework, etc.) from our study and similar studies from other countries (Membrive-Jiménez et al., 2020; Supervía and Bordás, 2020; Chodkiewicz et al., 2021). About 20.6% of students were under suicide risk from our study in Turkey, and their resilience to mental disorders is to be improved. Thus, they needed urgent support from families and governments. Women showed low-level coping with the pandemic situation with regard to mental health. Students’ development can be improved with social and economic support besides mental support. Students need financial support and improved relationships with colleagues and families. Protective factors improving individual adaptation and coping with trauma, tragedy, or extreme threats can enhance people’s resilience as buffers (Jojoa et al., 2021). Spiritual experiences and spiritual resources; believing in religious faith (Arslan and Yıldırım, 2021; Kar et al., 2021); social support from family, community, and university; sharing problems with others; cognitive reappraisal (Bourion-Bédès et al., 2021; Chodkiewicz et al., 2021; Jojoa et al., 2021); building healthy mental responses; responding effectively to crisis; learning to adapt to adversity (Jawad et al., 2020); living with family (Coppola et al., 2021; Deng et al., 2021); physical exercise for calming down (Kilani et al., 2020; Bourion-Bédès et al., 2021); improved dietary quality and sleeping score (Kilani et al., 2020; Kar et al., 2021); hoping the best (positive strategy); and staying busy for preventing thinking about the current situation (Bourion-Bédès et al., 2021; Chodkiewicz et al., 2021; Kar et al., 2021) are some ways to overcome the current stressful situation. Furthermore, cognitive-behavioral therapy delivered remotely (*via* digital health platforms telehealth) for both depression and anxiety through enhancing an individual’s awareness of own thoughts, feelings, and experiences and increasing personal resilience, requiring lifestyle changes (Aminoff et al., 2021; Surmai and Duff, 2022), hypnotic therapy, prolonged exposure therapy, stress inoculation therapy, group therapy, eye movement desensitization and reprocessing relaxation techniques, and pharmacological interventions are other improved treatment methods for the treatment of mental problems to reduce psychological problems in association with the pandemic (Cam et al., 2021).

This study was prepared as part of an international project (Rogowska et al., 2020), and some findings of the survey were used in another study (Ochnik et al., 2021) to compare with other countries in the second wave of the pandemic. The results of this study will guide university management, city administrators, social policy-makers, and families. This study fills the gap in the literature regarding the link between growing exposure to the COVID-19 pandemic from the first to the second wave of the pandemic and coronavirus-related stress, anxiety, depression, and PTSD among university students in Turkey.

### 5.1. Limitation of the study

Although this study found significant predictors for psychological problems, the main limitation regarding the cross-sectional design is not drawing any conclusions about the causality of the results. We do not have a baseline (pre-pandemic) measure, and longitudinal research is required in the future to verify the present findings. Self-reported measures may also include some sources of bias. In our study, 66.2% of participants were women, and it should be noted that in other studies, the gender ratios are close to each other. Different studies are needed, separately, only for Ph.D. students, only Master, or only undergraduate students, investigating PTSD, anxiety, perceived stress, depression level, and satisfaction with life during different waves of the pandemic, according to gender, financial situation, family situation (students on social assistance, students from single-parent families, students from disorganized families), whether they are athletes or not, etc.

## 6. Conclusion

Religious level, gender, and losing jobs as significant predictors of stress; relationship status, year of study, physical activities, COVID-19 symptoms, death of close relatives, and job loss as significant predictors of anxiety; and religious level, relationship status, year of study, physical activities, death of a close relative, job loss, and deterioration of economic status as significant predictors of depression were found. Also, religious level, gender, year of study, university, physical activities, COVID-19 symptoms, job loss, and deterioration of economic status were significant predictors of PTSD. These predictors found that attention should be given to economic improvement, female students, less exposure to COVID-19, more physical activities, and improved spiritual level to alleviate the effects of psychological problems. Perceived meaning of life and afterlife, believing in the good and fate by doing no harm, and protecting the interest of others and helping them, mediating adherence to preventive measures through moral principles, and compliance to authorities are helpful sides of religiosity, and religious organizations, healthcare organizations, and universities may work together. Religious support can be given to students in order to increase moral values, to shape their behavior, and to find solutions or alternatives to problems.

High anxiety, depression, PTSD prevalence, and declared having suicidal thoughts show that the second wave of the pandemic negatively affected the mental health of the students, and they need support from family and universities to recover with additional psychological and therapeutic support in order not to further intensify the disorders but to reduce or eliminate them. Governments can support last year and graduated students for job searches and professional development. Students living in villages, younger students, females, and single students were high-risk groups for mental problems.

## Data availability statement

The original contributions presented in this study are included in the article/supplementary material, further inquiries can be directed to the corresponding author.

## Ethics statement

This study was conducted according to the guidelines of the Declaration of Helsinki and approved by the local IRB: University Research Committee at the University of Opole, Poland, decision no.1/2020 and Bingöl University, decision no. 92342550/044/6137.

## Author contributions

IA contributed to the conceptualization of the study, formal analysis, methodology, supervision, visualization, and writing (original draft preparation). Both contributed to the data curation, investigation, project administration, resources, and review and editing, and have read and approved the final version of the manuscript.
